# Severe Growing-Up Phobia, a Condition Explained in a 14-Year-Old Boy

**DOI:** 10.1155/2014/706439

**Published:** 2014-12-21

**Authors:** Laurencia Perales-Blum, Myrthala Juárez-Treviño, Daniela Escobedo-Belloc

**Affiliations:** Universidad Autónoma de Nuevo León, Francisco I. Madero Avenue W and Gonzalitos Avenue, 64460 Monterrey, NL, Mexico

## Abstract

We present the clinical case of a 14-year-old boy with gerascophobia or an excessive fear of aging, who felt his body development as a threat, to the point where he took extreme measures to stop or otherwise hide growth. He had a history of separation anxiety, sexual abuse, and suffering bullying. He presented with anxious and depressive symptoms and food restriction, criticized his body image, had negative feelings towards the maturation process, suffered at the thought of being rejected, and was preoccupied with certain physical characteristics. We conducted an analysis of biological, psychological, and environmental factors and their possible interactions and established treatment with psychotherapy and fluoxetine. Because of the favorable results, this approach could be considered a good option in such cases.

## 1. Introduction

Gerascophobia is a fear of growing or aging [1]. Fear is an unpleasant emotion that occurs in response to a source of danger, whether real or imaginary, and has cognitive, behavioral, and physiological components [2]. It can also develop from the displacement of an emotion that arises from another environmental stressor (e.g., sexual abuse) [3].

The origin of phobias is multifactorial. It is caused by interactions between biological, psychological, and social/environmental factors. With regard to biology, children with an inhibited temperament who show apprehensive, hesitant, or distress reactions to new stimuli have a greater risk of developing clinical problems of anxiety [4, 5]. Psychologically, people with anxiety pay more attention to threat-related stimuli and have cognitive distortions that are consistent with anxiety [6]. Regarding environmental/social factors, it has been found that overprotective or very critical parents contribute to the development of anxiety [7]. Children can also learn to respond with fear through direct negative experiences [8].

## 2. Case Presentation

The patient is a 14-year-old adolescent whose problem started two and a half years ago due to an excessive fear of growing. He does not eat much because according to his own research food contains nutrients needed for physical development; in addition, he adopted a stooped posture to hide his height and began to distort his voice, using lower volume and higher pitch than usual, and he has also been searching the Internet to learn how not to ejaculate. He is greatly concerned with the development of secondary sexual characteristics. Every time he notices a physical change that indicates that he is growing, he feels fear and anxiety, to the point that has considered undergoing multiple surgeries to hide it. If people tell him that he is taller or older, he becomes extremely upset and cries. Due to the restriction in food intake, he has a weight loss of more than 12 kg. He is currently in the 25th percentile, according to the BMI for his age; however, he does not perceive any alterations in body image. Other discrepancies with regard to his physical appearance also coexist; he has Latin American features but his ideal of beauty is that of a Caucasian, “like Hollywood stars,” according to his own definition. He admires everything related to the United States. He has never been there but has plenty of information collected through the media. Although he believes that this fear is grossly excessive, he argues that the expectations that adults face are excessive: getting a partner, being independent, and having more responsibility and financial solvency. He also believes that once he reaches that age, he is more likely to get sick and die, all of which are very overwhelming.

Due to this problem, two years ago he consulted with a psychologist who treated this intense anxiety with cognitive behavioral therapy, gradually exposing him to the feared stimuli and using relaxation techniques; however, desensitization/habituation when facing his fears was not possible. After a year of 2-3 sessions per week his therapist together with his parents decided to refer him to our institution to continue his therapy, since it has a specialized area for treating adolescents and children.

With regard to his medical history, at age 5, he was diagnosed with separation anxiety disorder, for which he received psychotherapy once a week for a few months. The results were good and he achieved total remission. At age 6, he was sexually abused. A neighbor, 16 years of age, made him look at and touch his genitals and the neighbor also touched the patient. The parents noticed an emotional change in their child with a greater tendency to cry and later he also avoided going over to play with the neighbor's brother, who was his age, but it was not until he started treatment with the psychologist two years ago that he was able to talk about the sexual abuse he received during his childhood. He does not remember how long this went on, but he did recognize that the incident was repetitive. In sixth grade, he was a frequent victim of bullying (2-3 times a week). As for his family, his mother is an anxious person with dependent characteristics, while his father is rigid and often makes comments in a tone that the child sees as judging.

The rest of his development was normal. His mother had a normal pregnancy, planned and desired, with adequate prenatal care. There were no peri- or postnatal complications. The child was born at term by vaginal delivery with a birth weight of 3,950 g. He was discharged the next day with his mother and was breast-fed for 1 year and was weaned at 6 months. In the first months of life, he is described as calm. He was always under the care of his mother, who devoted all her time to the home. Psychomotor development was as expected: he sat at 6 months of age and walked and said his first words when he was 1 year old. The mother denies problems in movement coordination. He was toilet trained at the age of 2 years and 1 year later he also had nocturnal control. He slept with his parents until the age of 4 and in the same room until he was 6.

On arrival, the Birleson Depression Scale [9] was applied and the child had a score below the cutoff; the Anorectic Behavior Observation Scale (ABOS) [10] screening was positive; on the Body Shape Questionnaire (BSQ) [11, 12], a mild body image dissatisfaction was detected; a very high fear of maturity and interpersonal distrust (both in the 95th percentile) were identified in the Eating Disorder Inventory (EDI) [13, 14]. Accordingly, he had a significantly higher score in anxiety (4.6) and avoidance (3.5) in the Attachment Scale. On the Rorschach test, an elevated Armstrong and Loewenstein Trauma Content Index [15] and Perry and Viglione Critical Content [16] were found.

Regarding parental attitudes towards the problem, excessive care was provided by the mother, responding to changes in the patient's behavior with an attitude that corresponded to that of a much younger child: she would sing lullabies so he could sleep, chose the clothes that he would wear each day, combed his hair, and answered the questions that he was asked, not giving him the chance to answer. During consultations, she would cry when talking about the problems they faced as a family and openly expressed her despair and feeling that this was something that could not be fixed.

The father was angry with this scenario. He believed that his son was doing this to annoy or to get back at him for having been away such a long time due to his work. He tried to solve the problem by putting corrective posture belts on him and squeezing the curvature area tightly with his hands. The parent-child conflicts were constant until both chose to maintain a greater distance, further reducing communication. They even went so far as to avoid being in the same room.

As for treatment, since arrival at the institution and to date, psychotherapy has been provided. For one month and a half a crisis intervention model was used, attending with a frequency of 2-3 times per week. Retrospectively, a mentalization-based [17] approach has been used that has continued to this day with 50-minute sessions once a week.

From the beginning, the therapeutic alliance was prioritized because it is within a safe relational context where people can think, feel, and talk about traumatic events and thus reduce dissociative strategies; cognitive affect-regulation strategies identifying and naming emotions, being able to tolerate those which are dysphoric and favoring the pleasant ones have been modeled; the patient's affections have been validated, empathizing with him; situations are clarified, developing thoughts/feelings; feelings are related to the circumstances and emotions/reactions of others; exercises where he could practice the process of “reading minds”, which means perceiving and interpreting human behavior, considering mental states (Mentalization). Thus, an autobiographical narrative has been built where the meaning of mental states may reflect and explore past and present issues. Now the young man has a greater ability to understand that his reactions are based on something, which is the result of what happens to us in relation to others.

He was trained to know that what people think/feel is not obvious and other people may not know how he feels.

Fluoxetine 20 mg/day was started, increasing the dose to 40 mg/day after 6 weeks, obtaining an improvement of symptoms. He is currently seen with an upright posture and a natural tone of voice with no problems detected in his eating pattern. He has recovered 6 kg of weight; the ABOS scale is below the cut-off point for eating disorders. Mild body dissatisfaction persists in the BSQ and in the EDI great improvement is observed in almost all areas (obsession for thinness, body dissatisfaction, perfectionism, interpersonal distrust, and interoceptive awareness), except fear of maturity. The most obvious difference is in interpersonal distrust, which is currently in the 50th percentile. He attends school and has good performance. He has friends and participates in extracurricular activities (English classes). He does not show anxiety due to the presence of body hair or when he wears clothes that correspond to his age. He is able to imagine the future, living on his own and working as an actor, and this is an idea he likes; however, he continues to express a fear of commitment and responsibilities that he feels will be required of him in adult life.

Because the parents were overwhelmed and were not able to contain the patient, they were suggested to enter the family-to-family [18] course created by Dr. Joyce Bourland, founder of the National Alliance for the Mentally Ill, and they accepted this recommendation. This course is led and taught by trained family members of people living with mental illness and addresses biological/physical, psychological/emotional, and social/occupational aspects. The parents and the sister attended the 12 sessions, which are held once a week and have duration of 2.5 hours each.

Here, issues such as psychosocial disabilities and brain function are addressed; problem-solving skills and dealing with crises and relapses are learned; rehabilitation, expressing emotions at home, empathy, and techniques to improve communication are also covered, as well as WHO recommendations for addressing mental illness. At the end, his good performance/understanding was clearly seen; therefore, they were offered to be instructors of this movement.

Also, systemic family therapy was simultaneously carried out, lasting 60 minutes, once a week, for a total of 12 sessions. Therapy was aimed at improving family functioning, increasing the understanding and support among its members, unfocalizing the symptomatic patient, and increasing the skills of problem solving and coping techniques. Emphasis was placed on the strengths that the family has and the interactions that occur between its members. The mother tended to infantilize the patient, while the father tried to get away from him.

## 3. Discussion

Few cases of gerascophobia have been reported. We conducted various electronic searches and found only one article where two similar cases of fear of aging (in adults) are reported. These were conceptualized as “Dorian Gray syndrome,” characterized by dysmorphic-body symptoms, social phobia, and denial of the personality structure process toward maturity given by a reductionist view where too much emphasis is placed on the outward appearance. In the two patients mentioned, treatment was carried out by seeking to establish a climate of trust in the doctor-patient relationship and psychodynamic mediation was also done. The authors recommend intensive psychotherapy and, if necessary, the use of antidepressants and/or antipsychotics [19]. These cases have in common with our case the presence of anxiety, depression, self-criticism of body image, refusal to process maturation, grief at the thought of being rejected (related to attachment anxiety), and concern for their hair; however, in the “Dorian Gray Syndrome” the lack of hair is the concern, while in this case it was its appearance.

This patient showed an atypical eating behavior, although he did not meet criteria for an eating disorder, a finding that occurs in teenagers with difficulty in adjusting to the physical changes they are experiencing, which leads them to develop behaviors that produce a health risk. A history of sexual abuse is also common in young people with eating behavior problems. This could be a coping mechanism used to face a complex posttraumatic stress disorder or atypical depression [20]. In a study of 208 adolescent patients who were admitted to a psychiatric hospital, a subdiagnosis of significant concern with their weight and body image was found, although they did not meet the criteria for body dysmorphic disorder. Of the adolescents with these concerns, 22% had high scores of posttraumatic stress, dissociation, and worry or stress about sexuality. This group also shared high levels of anxiety, depression, and suicidality with the group suffering from dysmorphic disorder and eating disorder [21].

Specific phobia, such as gerascophobia, has a prevalence of 4–6%. It has been stated that several limbic structures contribute to the generation of this condition: the amygdala, the septum-hippocampus, and the hypothalamus-brain stem. These form part of a system that organizes the reactions to danger. Phobias and posttraumatic stress disorder are aspects of fear that could be considered conditioned. They involve processing discrete stimuli that can be considered a danger resulting in an exaggerated response. However, it is unusual to find a direct association between the catastrophic event and the phobic object [22]. This contrasts with the case described here, where the fear of developing physically could be attributed to a signal related to a danger of sexual abuse.

Trauma can be defined as an event that prevents the organization and storage of the experience at an explicit, reflective, and symbolic level. Instead, it is organized in intense emotional states and feelings that have no linguistic components. This dissociation is a fundamental part of the traumatic experience [23]. In the Rorschach test, elevated levels associated with posttraumatic stress and dissociative symptoms were found. In our patient, they reflect his concern and dissatisfaction with body image and the threat he experiences to his physical integrity.

Sexual abuse contributes to the development of anxiety disorders (such as phobias) because the victim develops a belief that the world is a dangerous place, where he/she has little control over what happens. In fact, greater prevalence of anxiety problems in people with a history of abuse has been seen [24, 25]. This explains why this boy had such a high score in the interpersonal distrust subscale of the EDI. One should also consider the fact that sexual abuse sensitizes the victim to the effects of subsequent traumatic exposures, which illustrates why the patient developed the fear to grow after bullying at school. Such awareness can be justified by the neural changes that stressful life events during childhood generate in the hypothalamus-pituitary-adrenal system, resulting in a persistent elevation of corticotropin releasing factor. The origins of the biological alterations that predispose to PTSD are not clear, but there is evidence that caregivers help modulate cortisol levels, a fact that supports the hypothesis that a lack of emotional availability generates a stress response in the child that in turn could trigger dysregulation that results in a greater risk for developing PTSD. It has even been postulated that early attachment experiences may be determinants of gene expression [23].

In adolescence, attachment insecurity, especially anxiousness, is a factor that determines the relationship between the various traumatic experiences of childhood, the onset of eating symptoms, and the impact that the media has on the development of body dissatisfaction [26–28]. The effect of attachment insecurity depends, at least in part, on the implicated affective-cognitive regulation strategies [29]. Furthermore, sexual abuse contributes to a feeling of shame and inadequacy and of incompetence and negative self-assessment [30].

Some hypothesize that an anxious temperament is a risk factor that predisposes to feeding problems, likely due to functional abnormalities in the limbic structures involved. Children with an inhibited temperament are very likely to have a highly reactive amygdala, which confers risk for developing separation anxiety disorder and social phobia. They are also more reactive to the expressions of signals that indicate internal states [23], which justifies why the patient was very likely to develop psychopathological symptoms when feeling rejected by peers. Feeding problems also reflect certain personality traits: cognitive restraint, resistance to change, rigidity, and tendency to obsessions [31]. The teenager described here has strong resistance and fear of body and social changes that accompany growth.

The body image is composed of a cognitive-affective component, one behavioral component, and one perceptual component. Within the behavioral component, the strategies are control and avoidance. When there is sexual abuse, the body may be associated with certain aspects of trauma and therefore develop avoidance of some body aspects [32], which is why this boy, unconsciously, seeks to prevent sexual maturation. Furthermore, in vulnerable individuals, having to leave their family to find their own niche and face emotional and sexual intimacy increases the feeling of a lack of control, which is a contributing factor to the fact that this boy refuses to grow [33]. He feels that the requirements of adult life are overwhelming; they are too complex.

Sexual abuse in addition to increasing the risk of posttraumatic stress disorder also increases twofold the risk of developing an eating disorder, without altering the perceptual component (as in anorexia). Our patient sees himself as a thin person, but he has body dissatisfaction, which is common in patients who have been victims of abuse [32]. It is believed that body dissatisfaction may be the factor that concatenates sexual abuse and feeding problems. Sexual abuse has a significant and lasting impact on identity, body image, self-regulation, and interpersonal function [28].

It was decided to use a mentalization-based therapy because, in victims of traumatic events, the capacity that enables them to make sense of their own and others' experiences [33] is inhibited [34]. Mentalization refers to the representational process where emotions/impulses are transformed into symbolic elements. This elaboration of the inner world and therefore of reflective capacity is a determinate factor to tolerate negative emotions and thus this factor prevents a discharge through an impulsive, excessive, or inappropriate behavior. It can be considered that there is a poor mentalization ability when the patient has a state of concreteness (in this case, the attempt to stop physical growth), without a greater reflective ability [35]. It is known that when a traumatic event occurs, the person's sense of basic security and his/her relationships with others are lost; distrust in the skills to face the future is generated and there is a loss in the ability to conceive mental states, particularly when intense emotional states are experienced, even to the point of conceiving that all that exists is the physical world. This creates a lack of flexibility that is known as psychic equivalence, where thoughts are perceived as reality. This prevents perceiving other perspectives. The ability to interact with others at a mental level is replaced by attempts to alter thoughts/feelings through action [36]. Phobias may be related to internal experiences, such as thoughts/feelings/fantasies/sensations. These are not based solely on fear but are also the result of negative predictions that are conditioned. Traumatized individuals come to avoid aspects of their normal lives, evading taking healthy risks, changes, or intimacy. It is common for them to have high prevalence of apprehension, due to a deficit in affect-regulation skills, and mentalization. This can be considered a defensive dissociative mechanism that is directed at intolerable feelings and experiences of trauma, preventing its meaning being understood. Thus, the previously neutral stimuli come to evoke in a sensorimotor manner the traumatic event with a great emotional burden. A verbal or conscious access to this memory is not possible; it is only evoked when there is a trigger. With the increase in mentalization/symbolization, traumatic memories are converted into narrative memories, ensuring their understanding and integration into the autobiographical memory [37].

Finally, it is important to provide adequate treatment to parents, who in response to trauma can cause the adolescent to have difficulty setting goals, ideas, and values, which are necessary to give a sense of continuity and cohesion [38].

## 4. Conclusion

In this case, biological (anxiety problems at an early age), psychological (interpersonal distrust), and environmental (exposure to trauma) risk factors were detected, which converged to result in an excessive fear of growing. Most mental problems rarely have a single cause, but a chain of events that involves genetic, environmental, social, and biological risk factors [28]. Because in the above process psychotherapy based on mentalization, attachment theoretical framework, and fluoxetine worked favorably, this could be considered a suitable strategy for similar symptoms.
